# Prognostic factors in patients with heart failure and sarcopenia: an observational retrospective study

**DOI:** 10.1186/s43044-024-00484-4

**Published:** 2024-04-29

**Authors:** Yasutaka Imamura, Atsushi Suzuki, Kazuho Kamishima, Kazuhito Suzuki, Junichi Yamaguchi

**Affiliations:** 1 Department of Cardiology, Rissho Koseikai Kosei Hospital, Tokyo, Japan; 2 Department of Cardiology, Tokyo Women’s Medical University, 8-1 Kawada-cho, Shinjuku-ku, Tokyo, 162-8666 Japan

**Keywords:** Brain natriuretic peptide, Ejection fraction, Heart failure, Prognostic factors, Sarcopenia

## Abstract

**Background:**

Heart failure (HF) prevalence increases with age, and sarcopenia is a poor prognostic factor in patients with HF. We aimed to evaluate the characteristics and prognostic factors in patients with HF and sarcopenia.

**Results:**

We retrospectively reviewed 256 consecutive patients admitted to our hospital for HF between May 2018 and May 2021, underwent dual-energy X-ray absorptiometry, and were diagnosed with sarcopenia. The primary endpoint was all-cause mortality. The prognoses and characteristics were evaluated and compared between patients with left ventricular ejection fraction (LVEF) < 50% (reduced LVEF, HF with reduced ejection fraction [HFrEF]) and those with LVEF ≥ 50% (preserved LVEF, HF with preserved ejection fraction [HFpEF]). 83 (32%) and 173 (68%) patients had HFrEF and HFpEF, respectively. The HFrEF group had fewer women, lower hypertension rates, higher ischemic heart disease rates, and brain natriuretic peptide (BNP) levels than did the HFpEF group. Kaplan–Meier analysis for all-cause death showed that the HFrEF group had a significantly worse prognosis than the HFpEF group [log-rank *p* = 0.002].

**Conclusions:**

In patients with HF and sarcopenia, older age, higher New York Heart Association (NYHA) class, BNP levels, and reduced LVEF were independent predictors of death after evaluation. During the treatment of patients with HF and sarcopenia, it is necessary to manage treatment with close attention to BNP and LVEF.

## Background

The incidence and number of patients with heart failure (HF) have been increasing globally [1, 2], with more than 40% of patients presenting with preserved left ventricular ejection fraction (LVEF) [3, 4]. Most patients with HF who are hospitalized with preserved LVEF are older adults [4, 5], with preserved LVEF as a common cause of HF owing to the progression of cardiac hypertrophy and myocardial fibrosis with aging, decreased ventricular compliance, and various complications such as renal failure and lung disease [6].

Sarcopenia, an age-related decrease in skeletal muscle mass and strength, has been previously associated with HF [7, 8]. The prevalence of sarcopenia among patients with HF is approximately 20%, which is higher than that among patients without HF [9]. The rates of sarcopenia among inpatients and outpatients with HF are 55% and 26%, respectively [10]. The coexistence of sarcopenia and HF is associated with decreased physical activity and increased mortality [11, 12]. Furthermore, sarcopenia is known to be involved in the exacerbation of HF [13].

LVEF plays a major role in hemodynamics. However, the relationship between LVEF and the prognosis of patients with HF remains controversial. According to prospective international data obtained by comprehensively evaluating and monitoring LVEF, patients with preserved LVEF have a lower risk of death than those with reduced LVEF [14]. However, previous reports show that the survival rate is not associated with LVEF [15, 16], and the prognosis does not differ between older male patients with HF and LVEF < 45% and LVEF > 45% [17]. Thus, the relationship between prognosis and LVEF in patients with HF and sarcopenia, which comprises a large number of elderly people, remains unclear.

In addition to older age, comorbidities have been associated with prognosis in older patients with chronic HF [18, 19]. Although the prognostic impact of several factors, such as age or LVEF, has been reported in cohorts of patients with HF, they have not been clearly defined in those with sarcopenia. Therefore, this study aimed to examine the differences in the characteristics and prognoses of patients with HF along with sarcopenia according to their LVEF and determine the prognostic factors.

## Methods

### Study population

This was a retrospective observational study conducted using data from 2,321 patients admitted to our hospital between May 2018 and May 2021. Finally, we included 256 patients meeting the diagnostic criteria for acute HF or exacerbation of chronic HF, who underwent rehabilitation and dual-energy X-ray absorptiometry (DXA), were diagnosed with sarcopenia, and subsequently underwent transthoracic echocardiography (TTE) in the analysis.

HF was defined using the Framingham criteria in the included patients [20]. If patients had multiple admissions, data from the first admission during the study period were used. Sarcopenia was diagnosed based on the 2019 definition of the Asian Working Group for Sarcopenia (AWGS) [21].

**Fig. 1:**
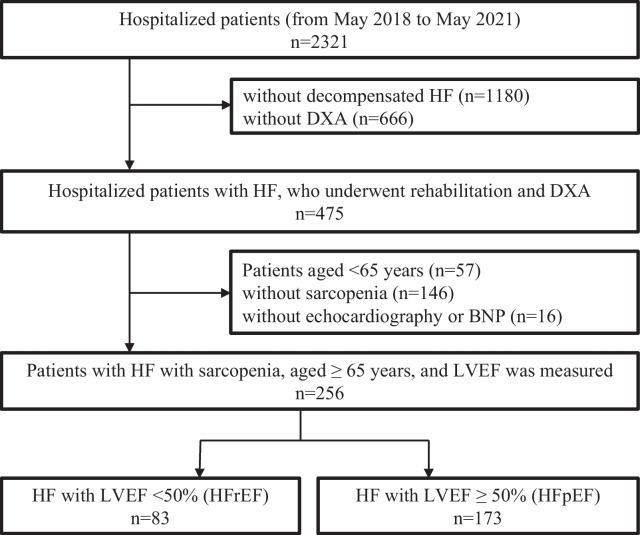
Patient selection flow chart for the study. *BNP* B-type natriuretic peptide; *DXA* dual-energy X-ray absorptiometry, *HF* heart failure, *HFpEF* heart failure with preserved ejection fraction, *HFrEF* heart failure with reduced ejection fraction, *LVEF* left ventricular ejection fraction

Specifically, the following criteria were used for diagnosing sarcopenia: age ≥ 65 years, grip strength < 28 kg for male patients and < 18 kg for female patients, walking speed < 1.0 m/s, and skeletal muscle mass index (SMI) measured by DXA < 7.0 kg/m^2^ and < 5.4 kg/m^2^ for male and female patients, respectively. Patients who could not undergo DXA and those with HF who did not undergo DXA or whose grip strength or walking speed could not be measured due to muscle weakness or dementia were excluded (Fig. 1). All the patients who underwent TTE (Vivid E9, S6; GE Healthcare Japan, Tokyo, Japan) were divided into two groups: those with LVEF < 50% (HF with reduced ejection fraction [HFrEF]) and those with LVEF ≥ 50% (HF with preserved ejection fraction [HFpEF]). The prognoses and characteristics of each group were evaluated and compared. Patients’ characteristics included age, sex, body mass index, SMI, New York Heart Association (NYHA) functional class, etiologies of heart disease, presence of comorbidities (hypertension, diabetes mellitus, and dyslipidemia), and smoking history.

### Ethics committee approval

This retrospective observational study was approved by the Ethics Committee of our hospital. The procedures followed were in accordance with the Declaration of Helsinki and the ethical standards of the Ethics Committee. The requirement for informed consent was waived owing to the retrospective nature of the study. We provided the study details to the patients and employed an opt-out method, clearly informing the patients of their right to decline enrolment.

### Prognostic evaluation

#### Primary endpoint

The primary endpoint of this study was all-cause mortality. Information on death was collected from patients’ medical records. Cardiovascular and cerebrovascular causes of death included HF, ventricular arrhythmia or sudden death, myocarditis, aortic disease, and stroke. Sudden death was defined as witnessed prodromal symptoms lasting < 24 h and the patient dying immediately at the location of identification or following successful resuscitation, primarily from cardiac arrest, but without neurological recovery.

#### Measurement of body composition using DXA

Body composition was measured by certified radiological technologists using whole-body DXA (Lunar iDXA; GE Healthcare, Madison, WI, USA). Appendicular SMI (ASM) was calculated via the sum of the muscle mass of the limbs, as measured by DXA. SMI was calculated using the following formula: ASM (kg) divided by the square of the height (m).

#### Blood sampling and TTE

Blood was collected during index hospitalization and used to measure the estimated glomerular filtration rate (eGFR) and low-density lipoprotein, brain natriuretic peptide (BNP), and glycated hemoglobin (HbA1c) levels. eGFR was estimated from the serum creatinine level using the abbreviated Modification of Diet in Renal Disease formula [22]. The BNP levels were measured using the BNP-JP chemiluminescent immunoassay (Abbott Japan, Chiba, Japan).

In addition, TTE was performed before patient discharge. LVEF, left ventricular end-diastolic diameter, and left ventricular end-systolic diameter were measured using the modified Simpson’s method.

### Statistical analysis

Each value is presented as a patient count or median with an interquartile range (IQR). We used the Chi-square test or Fisher’s exact test to compare categorical variables. The Wilcoxon–Mann–Whitney test was used to compare continuous variables between the two groups. The Kaplan–Meier method was used to compare the estimated cumulative survival rates between patients with HFrEF and those with HFpEF. We used the Cox proportional hazards model to identify the prognostic factors in patients with sarcopenia. Univariate and multivariate Cox regression analyses were used to estimate the relationship between baseline clinical characteristics and all-cause mortality. Clinical variables were chosen based on previously reported predictive clinical outcomes in HF, such as age, female sex, eGFR, albumin, and log plasma BNP levels [23], ischemic etiology, blood pressure, NYHA functional class, and use of beta-blockers and angiotensin-converting enzyme (ACE) inhibitors/angiotensin-receptor blockers (ARBs). The cutoff value of log BNP was based on the median level (2.5) in the Cox analysis for survival. Variables with *p* < 0.05 in the univariate analysis were included in the multivariate analysis. All data were analyzed using the IBM SPSS Statistics software (version 22.0; IBM Corp., Armonk, New York, USA).

## Results

### Patients’ characteristics

The data obtained from 256 patients with HF and sarcopenia who underwent TTE and had measurable LVEF were analyzed. A total of 173 (68%) patients had HFpEF and 83 (32%) had HFrEF. After the DXA evaluation, the patients were followed up for a median period of 138 days (IQR, 37–474). The duration of hospital stays of the 14 patients who died at discharge was 24 days (IQR: 18–69).

A comparison of the characteristics of patients with HFpEF and those with HFrEF did not show significant differences in age or body mass index. In addition, there were no differences in the rates of diabetes and hyperlipidemia between the two groups. However, the HFpEF group tended to be older (*p* = 0.076), have a lower rate of smoking history (*p* = 0.058), and have a significantly higher rate of hypertension (*p* = 0.002).

There was no difference in serum albumin and total cholesterol levels or eGFR between the two groups. However, the HFpEF group had a lower BNP level (210 mg/dL) than the HFrEF group (*p* < 0.001). Although there was no difference in the history of diabetes, the HbA1c level was higher in the HFrEF group than in the HFpEF group (*p* = 0.033). The median LVEF values were 40% and 62% in the HFrEF and HFpEF groups, respectively (*p* < 0.001).

Regarding the etiology of heart disease, fewer cases of ischemic heart disease and more cases of arrhythmia were observed in the HFpEF group than in the HFrEF group (Table 1). Arrhythmias included the types that can cause HF, such as atrial fibrillation and atrial tachycardia.

**Table 1:** Characteristics of heart failure patients with sarcopenia and reduced or preserved left ventricular ejection fraction

	All patients (*n* = 256)	HFrEF (*n* = 83)	HFpEF (*n* = 173)	*p* value
Age (years)	86 (79–91)	85 (78–89)	87 (80–92)	0.076
Sex (female) (%)	129 (50%)	33 (40%)	96 (55%)	0.018
BMI (kg/m^2^)	20.9 (18.4–22.9)	20.5 (17.8–22.7)	21.2 (18.7–23.1)	0.220
SMI (kg/m^2^)	5.1 (4.6–5.8)	5.1 (4.5–5.9)	5.1 (4.6–5.7)	0.780
NYHA functional class I/II/III/IV	15/34/102/105	7/6/29/41	8/28/73/64	0.055
	(6/13/40/41%)	(8/7/35/49%)	(5/16/42/37%)	
Etiology				0.001
Ischemic heart disease	87 (34%)	40 (48%)	47 (27%)	
Hypertensive heart disease	18 (7%)	3 (4%)	15 (9%)	
Dilated cardiomyopathy	3 (1%)	2 (2%)	1 (1%)	
Valvular heart disease	43 (17%)	17 (20%)	26 (15%)	
Arrhythmias	70 (27%)	13 (16%)	57 (33%)	
Other causes	35 (14%)	8 (10%)	27 (16%)	
Hypertension	132 (52%)	31 (37%)	101 (58%)	0.002
Diabetes mellitus	56 (22%)	20 (24%)	36 (21%)	0.552
Dyslipidemia	65 (25%)	24 (29%)	41 (24%)	0.369
Smoking history	96 (38%)	38 (46%)	58 (34%)	0.058
Serum albumin (g/dL)	3.2 (2.8–3.6)	3.2 (2.9–3.6)	3.2 (2.8–3.6)	0.359
Total cholesterol (mg/dL)	162 (133–194)	162 (134–194)	162 (132–194)	0.919
LDL cholesterol (mg/dL)	92 (73–119)	87 (73–120)	93 (72–119)	0.922
Triglyceride (mg/dL)	82 (66–115)	87 (71–117)	80 (64–112)	0.378
HbA1c (%)	5.9 (5.5–6.5)	5.9 (5.7–6.7)	5.9 (5.4–6.4)	0.033
eGFR (mL/min/1.73 m^2^)	46 (30–61)	48 (31–62)	44 (29–57)	0.476
BNP (pg/mL)	279 (131–627)	572 (210–823)	210 (106–454)	< 0.001
Log BNP	2.4 (2.1–2.8)	2.3 (2.0–2.7)	2.8 (2.3–2.9)	< 0.001
LVEF (%)	58 (45–64)	40 (34–45)	62 (58–65)	< 0.001
LVDd (mm)	48 (43–53)	54 (49–57)	46 (41–49)	< 0.001
LVDs (mm)	32 (28–39)	41 (38–46)	29 (26–33)	< 0.001
Medication at DXA evaluation				
ACE inhibitor or ARB	88 (34%)	31 (27%)	57 (33%)	0.488
Beta-blocker	85 (33%)	25 (30%)	60 (35%)	0.450
MRA	32 (13%)	10 (12%)	22 (13%)	0.880
Diuretic	97 (38%)	36 (43%)	61 (35%)	0.210
Statin	60 (24%)	20 (24%)	40 (23%)	0.823
Medication at discharge	(*n* = 242)	(*n* = 78)	(*n* = 164)	
ACE inhibitor or ARB	128 (53%)	43 (55%)	85 (52%)	0.631
Beta-blocker	93 (38%)	30 (38%)	63 (38%)	0.994
MRA	63 (26%)	26 (34%)	37 (23%)	0.065
Diuretic	161 (67%)	61 (78%)	100 (61%)	0.008
Statin	78 (32%)	28 (36%)	50 (30%)	0.363

*ACE* angiotensin-converting enzyme, *ARB* angiotensin-receptor blocker, *BMI* body mass index, *BNP* B-type natriuretic peptide, *DXA* dual-energy X-ray absorptiometry, *eGFR* estimated glomerular filtration rate, *HbA1c* glycated hemoglobin, *HFpEF* heart failure with preserved ejection fraction, *HFrEF* heart failure with reduced ejection fraction, *LDL* low-density lipoprotein, *LVDd* left ventricular end-diastolic diameter, *LVDs* left ventricular end-systolic diameter, *LVEF* left ventricular ejection fraction, *MRA* mineralocorticoid receptor antagonist, *NYHA* New York Heart Association, *SMI* skeletal muscle mass index

At the time of DXA scanning, there were no between-group differences in the administration rates of beta-blockers, ACE inhibitors or ARBs, mineralocorticoid receptor antagonists (MRAs), and diuretic drugs. At discharge, more than half of the patients had received ACE inhibitors or ARBs. The rate of oral diuretic administration was significantly higher in patients with HFrEF than in those with HFpEF (*p* = 0.008).

#### Outcomes

**Fig. 2:**
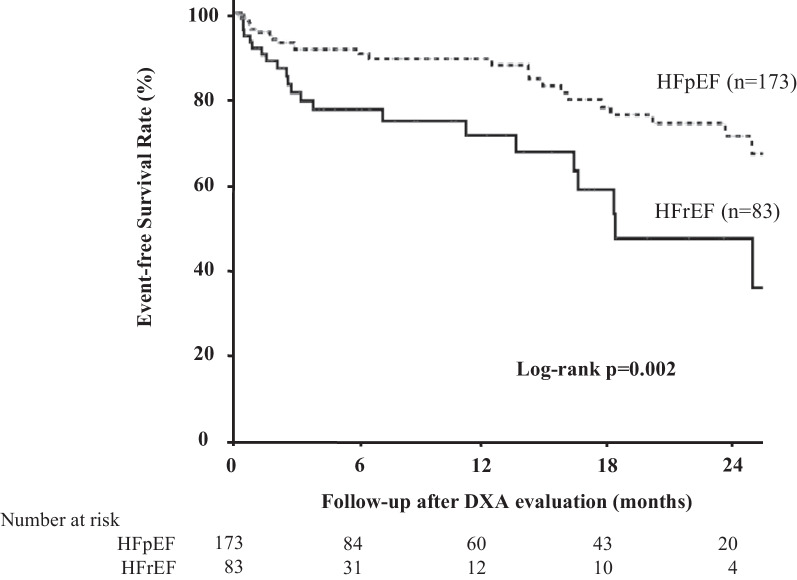
Comparison of prognosis between the HFrEF (EF < 50%) and HFpEF (EF ≥ 50%) groups. Kaplan–Meier analysis shows that the HFpEF group had a significantly better survival rate than the HFrEF group (log-rank *p* = 0.002). *DXA* dual-energy X-ray absorptiometry, *HFpEF* heart failure with preserved ejection fraction, *HFrEF* heart failure with reduced ejection fraction

Twenty-five deaths (14%) in the HFpEF group and 22 (27%) in the HFrEF group were observed. Regarding the causes of mortality, 18 (38%), 12 (26%), and 5 (10%) patients died of HF, infectious disease, and malignant disease, respectively. Among patients who died, there were no differences in the rates of cardiovascular or cerebrovascular causes of death between the HFrEF and HFpEF groups (Table 2). The Kaplan–Meier analysis showed that the HFpEF group had a better prognosis than that of the HFrEF group (log-rank *p* = 0.002) (Fig. 2). In the HFrEF and HFpEF groups, the survival rates at 6, 12, and 24 months were 77% and 90%; 71% and 89%; and 71% and 46%, respectively.

**Table 2:** Causes of death among HFrEF and HFpEF patients

	All patients (*n* = 256)	HFrEF (*n* = 83)	HFpEF (*n* = 173)	*p*-value
All-cause death	47 (18%)	22 (27%)	25 (14%)	0.020
Cardiovascular or cerebrovascular cause	24 (51%)	13 (59%)	11 (44%)	0.471
Heart failure	18 (38%)	10 (45%)	8 (32%)	
Ventricular arrhythmia or sudden death	3 (6%)	2 (9%)	1 (4%)	
Myocarditis	1 (2%)	1 (5%)	0 (0%)	
Aortic dissection	1 (2%)	0 (0%)	1 (4%)	
Stroke	1 (2%)	0 (0%)	1 (4%)	
Other cause	23 (49%)	9 (41%)	14 (56%)	
Infection	11 (23%)	5 (23%)	6 (24%)	
Malignancy	5 (10%)	2 (9%)	3 (12%)	
other non-cardiac cause	3 (6%)	1 (5%)	2 (8%)	
Unknown	4 (9%)	1 (5%)	3 (12%)	

HFpEF heart failure with preserved ejection fraction; HFrEF heart failure with reduced ejection fraction

#### Prognostic factors

The univariate Cox analysis for survival in patients with HF and sarcopenia showed that age ≥ 85 years (hazard ratio [HR] 2.316, 95% confidence interval [CI]: 1.270–4.227, *p* = 0.006), higher NYHA functional class (HR 1.543 per class, 95% CI: 1.061–2.244, *p* = 0.023), reduced LVEF (HR 2.469 per class, 95% CI: 1.383–4.405, *p* = 0.002), and log BNP level ≥ 2.5 (HR 3.454 per class, 95% CI: 1.789–6.668, *p* < 0.001) had significant predictive values.

The multivariate analysis showed that reduced LVEF had a significant predictive value for mortality (HR 2.066, 95% CI: 1.110–3.861, *p* = 0.022). Other independent predictors for survival included age ≥ 85 years (HR 2.435, 95% CI: 1.310–4.526, *p* = 0.005), higher NYHA functional class (HR 1.635, 95% CI: 1.100–2.429, *p* = 0.015), and log BNP level ≥ 2.5 (HR 2.885, 95% CI: 1.487–5.596, *p* = 0.002; Table 3).

**Table 3:** Univariate and multivariate analyses related to prognosis

	Univariate analysis			Multivariate analysis		
	HR	95% CI	*p*	HR	95% CI	*p* value
Male sex	0.944	0.532–1.673	0.843			
Age ≥ 85 years	2.316	1.270–4.227	0.006	2.435	1.310–4.526	0.005
NYHA functional class	1.543	1.061–2.244	0.023	1.635	1.100–2.429	0.015
Ischemic etiology	1.318	0.738–2.354	0.350			
HFrEF vs. HFpEF	2.469	1.383–4.405	0.002	2.066	1.110–3.861	0.022
Systolic BP per 10 mmHg decrease	1.107	0.995–1.233	0.063			
Albumin < 3.8 g/dL	2.341	0.922–5.943	0.074			
eGFR per 10 mL/min/1.73 m^2^ decrease	1.135	0.999–1.289	0.050			
Log BNP ≥ 2.5	3.454	1.789–6.668	< 0.001	2.885	1.487–5.596	0.002
Beta-blocker	0.929	0.495–1.742	0.818			
ACE inhibitor or ARB	0.719	0.379–1.367	0.315			

*ACE* angiotensin-converting enzyme, *ARB* angiotensin-receptor blocker, *BNP* B-type natriuretic peptide, *BP* blood pressure, *eGFR* estimated glomerular filtration rate, *CI* confidence interval, *HFpEF* heart failure with preserved ejection fraction, *HFrEF* heart failure with reduced ejection fraction, *HR* hazard ratio, *NYHA* New York Heart Association

## Discussion

In the present study, we evaluated the characteristics and prognoses of patients with HF and sarcopenia. The results indicated that patients with HFpEF were more likely to be women, have arrhythmia as the etiological cause of HF, and have lower BNP levels than patients with HFrEF. Patients with HFrEF had a significantly worse prognosis than those with HFpEF. Other independent prognostic factors included older age, a higher NYHA functional class, and higher BNP levels.

Few studies have accurately defined sarcopenia. Some studies were conducted using a clear diagnosis of sarcopenia with muscle strength, physical function, and skeletal muscle mass based on DXA results and the AWGS definition, indicating that a high BNP level is associated with sarcopenia in patients with diabetes [24, 25]. However, few studies on the prognosis of patients with or without HF have been conducted, including a limited number of studies on patients with sarcopenia that defined sarcopenia based on DXA or bioelectrical impedance analysis results, grip strength, and walking speed [5, 24, 26]. A total of 256 patients with HF who were diagnosed with sarcopenia according to the AWGS definition were analyzed in the present study, which is a relatively large number of patients examined in this research context to date.

Previous studies of cardiac function, body composition, and prognosis have demonstrated that HFrEF reduces axial muscle mass and is an independent predictor of mortality. In addition, there is a significant inverse correlation between skeletal muscle mass and N-terminal pro-brain natriuretic peptide (NT-proBNP) levels. The skeletal muscle mass is higher, and the NT-proBNP level is lower in patients with HFpEF than in those with HFrEF [27].

In the Japanese Cardiac Registry of Heart Failure in Cardiology study of patients with HF, 26% had HFpEF and 81% of them were 65 years or older [15]. HFpEF is common among older people because it is associated with various complications such as renal failure, lung disease, and reduced ventricular compliance. In the present study, 68% of the patients had HFpEF, which was higher than the proportion of patients with HFrEF. Reportedly, there is no difference in the prognosis of older people with different ejection fractions. However, the results of the present study showed that patients with ejection fraction < 50% had poor prognoses.

In general, older patients with HF should be treated according to the current HF guidelines [28]. However, in the present study, ACE inhibitors/ARBs (53%), beta-blockers (38%), and MRAs (26%) were used insufficiently at discharge, particularly in patients with HFrEF and sarcopenia (55%, 38%, and 34%, respectively). From the results of the Change the Management of Patients with HF (CHAMP-HF) registry, older age and renal dysfunction have been reported to be associated with lower prescription rates of HF medications (ACE inhibitors/ARBs/angiotensin-receptor-neprilysin inhibitors, beta-blockers, and MRAs) [29]. Patients with sarcopenia are cautious when taking medications because of concerns regarding adherence and comorbidities. In the present study, many patients with HF and sarcopenia had renal dysfunction and were older, which may have contributed to a lower prescription rate of medications. Among our study patients, low rates of HF medication use might have resulted in a worse prognosis in HFrEF than in HFpEF. In addition to HF likely occurring with other comorbidities associated with disabilities and prognosis [30, 31], the pharmacological treatment of HF in older adults remains a challenge.

BNP is a useful predictor of the prognosis of cardiovascular events in the general population [32]. The relationship between sarcopenia and BNP levels has been reported in previous studies. The prevalence of sarcopenia is reportedly high among patients with diabetes without HF who have high BNP levels (cutoff value, 27.3 pg/mL) [24]. Furthermore, patients with HF and those who underwent weight loss have high BNP levels and thin epicardial adipose tissue [33]. In addition, the sarcopenia score has been reported to be a poor prognostic factor for HF, and the prognosis is worse when the BNP level is high, which is consistent with our results [26]. BNP is a powerful prognostic indicator for HF at any disease stage, as well as for sarcopenia.

Hanatani et al. reported that in patients with chronic kidney disease, a high sarcopenia score was a poor prognostic factor, and patients with high sarcopenia scores had significantly lower eGFR values than those with low sarcopenia scores [34]. In this study, the median eGFR value was reduced (median eGFR: 46, IQR 30–61 mL/min/1.73 cm^2^) among patients with HF and sarcopenia. Although a lower eGFR tended to be associated with mortality in the Cox analysis, renal dysfunction is known to be associated with a poor prognosis in patients with HF. According to these data, sarcopenia signifies that skeletal muscle atrophy coexisting with HF is closely related to kidney disease and may lead to the progression of cardiovascular diseases.

This study had some limitations. The study participants were patients with HF hospitalized at a single center. Consequently, these results are not generalizable to groups with dissimilar demographics. The small sample size was insufficient to examine the other contributing factors associated with prognosis. Selection bias may have also been present, as we excluded patients on whom DXA could not be performed. Although DXA is widely used to diagnose sarcopenia, daily living activities were not assessed in all patients in this study. In addition, not all known prognostic factors may have been accurately measured, and confounding factors may not have been well-controlled because of the nature of our retrospective observational study. Nevertheless, the results of this study suggest that several risk factors, including reduced LVEF, could provide prognostic information for patients with HF and sarcopenia. There is a need to develop treatment strategies, including appropriate medications or rehabilitation, to improve prognosis among patients with HF and sarcopenia with risk factors.

## Conclusions

The present study showed that patients with HF and sarcopenia with reduced LVEF had worse prognoses than those with preserved LVEF. Furthermore, among patients with HF and sarcopenia, those with older age, higher NYHA class, and log BNP level ≥ 2.5 had worse prognoses. Accordingly, careful management should be considered for patients with reduced LVEF and high BNP levels. Among patients with HF and sarcopenia, those who have poor prognoses can be identified based on LVEF or BNP, and effective approaches to improve prognosis should be explored.

## Data Availability

All data generated or analyzed during this study have not been published but can be obtained by contacting the authors.

## Abbreviations

ACE: Angiotensin-converting enzyme
ARBs: Angiotensin-receptor blockers
ASMI: Appendicular SMI
AWGS: Asian Working Group for Sarcopenia
BNP: Brain natriuretic peptide
CHAMP-HF: Change the Management of Patients with HF
CI: Confidence interval
DXA: Dual-energy X-ray absorptiometry
eGFR: Glomerular filtration rate
HbA1c: Glycated hemoglobin
HF: Heart failure
HFpEF: Heart failure with preserved ejection fraction (
HFrEF: Heart failure with reduced ejection fraction
HR: Hazards ratio
IQR: Interquartile range
LVEF: Left ventricular ejection fraction
MRAs: Mineralocorticoid receptor antagonists
NT-proBNP: N-terminal pro-brain natriuretic peptide
NYHA: New York Heart Association
SMI: Skeletal muscle mass index
TTE: Transthoracic echocardiography
