# Correction: Prognostic factors in patients with heart failure and sarcopenia: an observational retrospective study

**DOI:** 10.1186/s43044-024-00521-2

**Published:** 2024-07-08

**Authors:** Yasutaka Imamura, Atsushi Suzuki, Kazuho Kamishima, Kazuhito Suzuki, Junichi Yamaguchi

**Affiliations:** 1 Department of Cardiology, Rissho Koseikai Kosei Hospital, Tokyo, Japan; 2 Department of Cardiology, Tokyo Women’s Medical University, 8-1 Kawada-Cho, Shinjuku-Ku, Tokyo, 162-8666 Japan

**Correction: The Egyptian Heart Journal (2024) 76:52** 10.1186/s43044-024-00484-4

Following publication of the original article [1], the authors identified the errors listed below.

1. **Section—Results, Subsection—Patients’ characteristics, Paragraph—2**:
  “However, the HFpEF group tended to be older (*p* = 0.076), have a higher rate of smoking history (*p* = 0.058), and have a significantly higher rate of hypertension (*p* = 0.002).” needs to be changed to “However, the HFpEF group tended to be older (*p* = 0.076), have a lower rate of smoking history (*p* = 0.058), and have a significantly higher rate of hypertension (*p* = 0.002).”
  **Text after correction**:
  A comparison of the characteristics of patients with HFpEF and those with HFrEF did not show significant differences in age or body mass index. In addition, there were no differences in the rates of diabetes and hyperlipidemia between the two groups. However, the HFpEF group tended to be older (*p* = 0.076), have a lower rate of smoking history (*p* = 0.058), and have a significantly higher rate of hypertension (*p* = 0.002).
2. **Table** **2**, **Row—Infection, Column—All patients (n = 256)**:
  “12 (26%)” needs to be changed to “11 (23%)”.
  **Text after correction**:

**Table 2:** Causes of death among HFrEF and HFpEF patients

	All patients (n = 256)	HFrEF (n = 83)	HFpEF (n = 173)	p-value
All-cause death	47 (18%)	22 (27%)	25 (14%)	0.020
Cardiovascular or cerebrovascular cause	24 (51%)	13 (59%)	11 (44%)	0.471
Heart failure	18 (38%)	10 (45%)	8 (32%)	
Ventricular arrhythmia or sudden death	3 (6%)	2 (9%)	1 (4%)	
Myocarditis	1 (2%)	1 (5%)	0 (0%)	
Aortic dissection	1 (2%)	0 (0%)	1 (4%)	
Stroke	1 (2%)	0 (0%)	1 (4%)	
Other cause	23 (49%)	9 (41%)	14 (56%)	
Infection	11 (23%)	5 (23%)	6 (24%)	
Malignancy	5 (10%)	2 (9%)	3 (12%)	
other non-cardiac cause	3 (6%)	1 (5%)	2 (8%)	
Unknown	4 (9%)	1 (5%)	3 (12%)
3. **Table** **3**
  “eGFR per 10 mL/min/1.73 cm^2^ decrease” needs to be changed to “eGFR per 10 mL/min/1.73 m^2^ decrease”.
  “Albumin < 10 mmHg/dL” needs to be changed to “Albumin < 3.8 g/dL”
  **Text after correction**:

**Table 3:** Univariate and multivariate analyses related to prognosis

	Univariate analysis			Multivariate analysis		
	HR	95% CI	p	HR	95% CI	p-value
Male sex	0.944	0.532–1.673	0.843			
Age ≥ 85 years	2.316	1.270–4.227	0.006	2.435	1.3104.526	0.005
NYHA functional class	1.543	1.061–2.244	0.023	1.635	1.100–2.429	0.015
Ischemic etiology	1.318	0.738–2.354	0.350			
HFrEF vs. HFpEF	2.469	1.383–4.405	0.002	2.066	1.110–3.861	0.022
systolic BP per 10 mmHg decrease	1.107	0.995–1.233	0.063			
albumin < 3.8 g/dL	2.341	0.922–5.943	0.074			
eGFR per 10 mL/min/1.73 m^2^ decrease	1.135	0.999–1.289	0.050			
Log BNP ≥ 2.5	3.454	1.789–6.668	< 0.001	2.885	1.487–5.596	0.002
Beta-blocker	0.929	0.495–1.742	0.818			
ACE inhibitor or ARB	0.719	0.379–1.367	0.315

The original article [1] has been corrected.
